# The Involvement of Amino Acid Side Chains in Shielding the Nickel Coordination Site: An NMR Study

**DOI:** 10.3390/molecules181012396

**Published:** 2013-10-08

**Authors:** Serenella Medici, Massimiliano Peana, Valeria Marina Nurchi, Maria Antonietta Zoroddu

**Affiliations:** 1 Department of Chemistry and Pharmacy, University of Sassari, Via Vienna 2, 07100, Sassari, Italy; E-Mails: sere@uniss.it (S.M.); peana@uniss.it (M.P.); nurchi@unica.it (V.M.N.); 2 Department of Chemical and Geological Sciences, University of Cagliari, Cittadella Universitaria, I-09042 Monserrato, Cagliari, Italy

**Keywords:** nuclear magnetic resonance spectroscopy, NMR structure, structural models, nickel peptide complexes, amino acid side chains

## Abstract

Coordination of proteins and peptides to metal ions is known to affect their properties, often by a change in their structural organization. Side chains of the residues directly involved in metal binding or very close to the coordination centre may arrange themselves around it, in such a way that they can, for instance, disrupt the protein functions or stabilize a metal complex by shielding it from the attack of water or other small molecules. The conformation of these side chains may be crucial to different biological or toxic processes. In our research we have encountered such behaviour in several cases, leading to interesting results for our purposes. Here we give an overview on the structural changes involving peptide side chains induced by Ni(II) coordination. In this paper we deal with a number of peptides, deriving from proteins containing one or more metal coordinating sites, which have been studied through a series of NMR experiments in their structural changes caused by Ni(II) complexation. Several peptides have been included in the study: short sequences from serum albumin (HSA), Des-Angiotensinogen, the 30-amino acid tail of histone H4, some fragments from histone H2A and H2B, the initial fragment of human protamine HP2 and selected fragments from prion and Cap43 proteins. NMR was the election technique for gathering structural information. Experiments performed for this purpose included 1D ^1^H and ^13^C, and 2D HSQC, COSY, TOCSY, NOESY and ROESY acquisitions, which allowed the calculation of the Ni(II) complexes structural models.

## 1. Introduction

It is well known that metal binding in a peptide or a protein may induce organization of amino acid side chains around the coordination site. It has been suggested that metal-induced sequence-specific conformational ordering of amino acid side chains [1] can be a powerful feature for the design of bioactive peptides as it can increase their stability by creating an axial hydrophobic fence that can shield them from the attack of water molecules [2]. This can also be true for protein metal complexes, especially when a specific side chain organization around the metal produces active sites for biological processes.

Actually, the stability of metal complexes with proteins or peptides is regulated by many factors and obviously this is of fundamental importance for biological processes where a metal-to-protein interaction is involved, ranging from metal toxicity to metal homeostasis and many others [3,4].

**Scheme 1:**
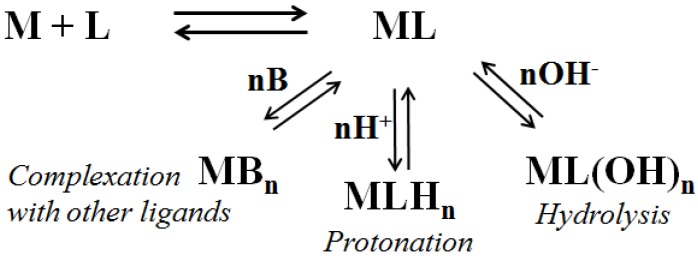
Side reactions that can decrease the stability of [ML] complex. Charges have been omitted for simplicity.

Factors determining a complex stability include the kind of metal ion, donor types, formation of five- or six-membered chelating rings or macrocycles, pH, competition with other ligands. The sum of all these effects is reflected in the stability constant for the complex [5]. Along the formation of the complex, a series of side reactions can undermine the stability of the coordinated species itself, such as competition with other ligands (like water or other small coordinating molecules present in the cellular environment), protonation and/or hydrolysis equilibria, *etc*. (see Scheme 1). Every factor hindering these side reactions is able to stabilize the complex, thus prolonging its lifetime: the organization of side chains around the central ion seems to be one of them.

In fact, during our research work and in the related literature, we encountered different cases of such behaviour when a metal ion like Ni(II) was able to form square planar complexes with a protein or its model peptides, and to induce a conformational change in its structures. In all cases a histidine residue was present, acting as an anchoring site for the metal; the experimental pH was normally kept at values allowing the formation of square planar diamagnetic species suitable for NMR investigation. Indeed, this is a very useful technique that allows calculating a model of the coordination site in solution when X-ray crystal structures are not obtainable, as to the best of our knowledge no such structure of a nickel complex with a histidine-containing peptide has so far been reported.

A combination of mono- and two-dimensional, mono- and multinuclear experiments is normally used; NOESY and ROESY are especially valuable since they allow the transformation of ^1^H-^1^H cross-correlations into upper distance limits used to build up the structural models, in which the arrangement of the side chains becomes clearly visible.

In this review we will discuss the structural changes and the involvement of amino acid side chains in the formation of Ni(II) complexes with peptides deriving from proteins where a histidine site is present. The examined peptides do not always bear the histidine residue in the same position: in some cases it is very close to the N-terminus, so that an involvement of the terminal NH_2_- group in the complex formation is expected; in other cases it is located in the middle of the peptide chain, or anyway quite far from the N-terminus, so that the coordination mode comprises only deprotonated amidic nitrogens from the backbone together with the N_Im_ donor.

## 2. Results and Discussion

### 2.1. Albumin

A pioneering investigation was carried out in 1984 on the N-terminal 24-residues peptide (NH_2_-D_1_A_2_H_3_K_4_S_5_E_6_V_7_A_8_H_9_R_10_FKDLGEENFKALVL) from human serum albumin (HSA), containing a specific binding site for Ni(II). Only mono-dimensional ^1^H- and ^13^C-NMR spectra were recorded on a 360 MHz and a 250 MHz spectrometer. Selective or multiple proton-decoupling techniques were used to assign the resonances for both free and Ni-bound peptides, with the help of the pH-dependent chemical shifts, the determination of the characteristic pK values, and the comparison with the minimal peptide model DAH-N-dimethylamide [6]. The series of experiments, carried out at pH higher than 8.00, showed that a pentacoordinated complex is formed, with the involvement in the coordination of the -NH_2_ terminal group, the imidazole nitrogen of the histidine residue in the third position (His_3_), two deprotonated amidic nitrogens from the backbone (belonging to Ala_2_ and His_3_ residues), together with the carboxylate moiety from aspartyl (Asp_1_) residue in the apical position, giving a {N_Im_, 2N^−^, NH_2_, O^−^} species. 

Furthermore, also Ser_5_, Val_7_, and Arg_10_ residues showed perturbations upon metal binding, while changes for Lys_4_ were not detected in the spectra. Thus, it appeared that complexation of Ni(II) affected as well some residues close to the metal, not by involving them directly into the binding, but either via direct through space effects or via a local conformational change.

**Figure 1:**
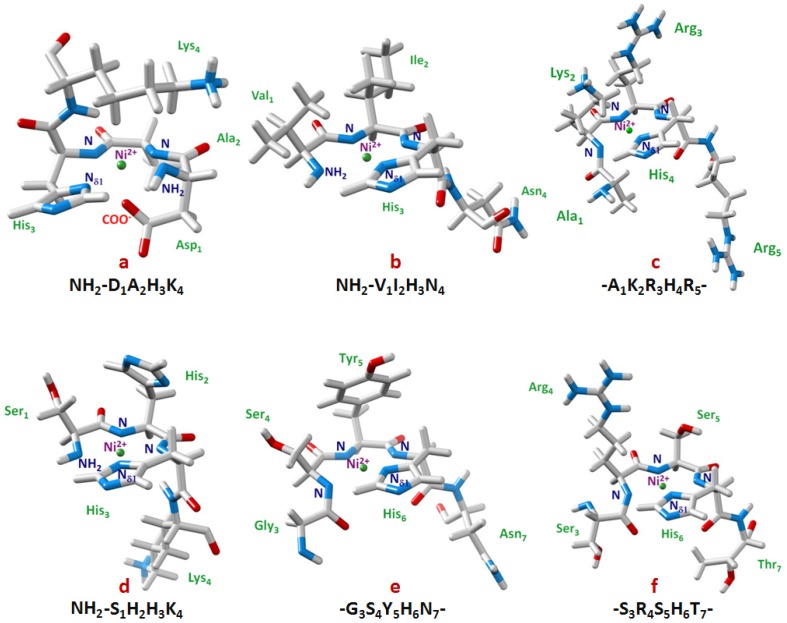
3D structural models for Ni(II) complexes in a square based pyramidal geometry {N_Im_, 2N^−^, NH_2_, O^−^} with NH_2_-DAHK- fragment from human serum albumin (**a**), and in a square planar mode with: (**b**) NH_2_-VIHN- from Des-Angiotensinogen {N_Im_, 2N^−^, NH_2_}, (**c**) -AKRHR- from histone H4 {N_Im_, 3N^−^}, (**d**) NH_2_-SHHK from histone H2A {NH_2_, N_Im_, 2N^−^}, (**e**) -GSYHN- from prion protein {N_Im_, 3N^−^}, (**f**) -SRSHT- from Cap43 protein {N_Im_, 3N^−^}. Ni(II) is reported in green and the amino acids according to the chemistry of their atoms: in gray (hydrogen and carbon), red (oxygen), and blue (nitrogen). The models **a**, **b**, **d** and **e** were obtained by molecular modeling and computational chemistry software HyperChem^(tm)^ 8.0.7 [10], models **c** and **f** by simulated annealing program DYANA [11]. Molecular graphics and analyses were performed with the UCSF Chimera package [12].

It was only ten years later that Lys_4_ clearly appeared to be involved in nickel binding to the protein. This fact has been reported in a successive study on defatted bovine (BSA) and human (HSA) serum albumins, being the two proteins different in the second residue, where a threonine in position 2 is located in the place of an alanine, and an extra histidine in position 59 of the bovine albumin [7]. As the progress in sciences had run fast and powerful high-field equipments had developed meanwhile, the study was carried out through mono- and two-dimensional ^1^H-NMR on a 500 MHz and a 600 MHz machine, by involving the whole protein instead of a single fragment or domain. In spite of its 66.5 kDa of weight, several regions of the protein are mobile enough to give rise to well resolved ^1^H-NMR resonances. This allowed for the assignment of the signals of the first N-terminal three amino acids of BSA, HSA, rat serum albumin and porcine serum albumin, when the use of high resolution ^1^H-NMR spectroscopy to study metal binding in proteins was beginning to be used [8,9]. Thus, by the combination of COSY, DQF-COSY, NOESY and TOCSY spectra, it was possible to show that, together with Asp_1_, Ala_2_ (or Thr_2_) and His_3_, also Lys_4_ was in some way involved in Ni(II) coordination at pH 7.67. In fact, the appearance of a new spin system for Lys_4_ was clearly evident after the addition of 1.0 equivalent of nickel. The complex again seemed to adopt a {N_Im_, 2N^−^, NH_2_, O^−^} coordination mode, involving the -NH_2_ terminal group, the imidazole N_δ_ from His_3_, two amide nitrogens from the backbone (Ala_2_ and His_3_), and a weak axial binding with the carboxylate side chain from Asp_1_ (Figure 1a).

The involvement of Lys_4_ was inferred from the difference in chemical shifts observed upon coordination. This affected the signals of γ, δ and ε protons of the Lys residue in Ni-HSA. The order of their shifts correlated well with ring-current calculations based on the geometry observed in the crystal structure of a copper complex with a short peptide containing both a histidine and a lysine residue, CuGHK, where the Lys side chain lies over the His ring [13].

Although the magnitudes of the predicted shifts did not correspond exactly to the experimental data, the relative ordering of the shifts (ε < δ < γ) was correct. The directional nature of the ring current shift [14], along with the other structural constraints of the complex, pointed strongly towards the proposed arrangement of Lys and His side chains. The pattern of the coordination shifts for the Lys residue after Ni(II) binding (ε < δ < γ) also suggested that its εNH_2_ group was not bound directly to nickel. While different cross relaxation were recorded by NOESY experiments, no contacts between Lys Hε and His Hε were observed, meaning that their distance was considerably large, and this may be related to the mobility of the side chains with respect to each other.

This was the first time the effects of nickel on a Lys residue of intact albumin had been reported and it was suggested that the conformation of this side chain may be crucial to the recognition of the Ni-HSA complex as the antigenic determinant in the case of nickel allergy. Furthermore, the fact that this effect had been recorded only in the whole protein and not in its 24-amino acid model underlines the importance of studying the entire protein to have a proper evaluation of the interaction between a metal and its biological targets; this aspect has been clarified lately with a comparative study on short peptides derived from albumins and their parent molecules [15]. It was then shown that while qualitative details of interaction (spectra and structures of complexes, order of reactions) could be reproduced, the quantitative parameters (stability and rate constants) could not. The N-terminal site of HSA, in fact, is much more similar to BSA than to short peptides reproducing the HSA sequence.

### 2.2. Des-Angiotensiogen

The influence of side chains in the formation of Ni(II) complexes started to become clear by studying the albumin protein, but it was with the work of Bal [2], shortly after, that the stability of the complex was put in close relation with the shielding effect a side-chain organization around the metal ion may offer against destabilizing factors, like the attack of water molecules. The protein under investigation was Des-Angiotensinogen and, in particular, its N-terminal histidine containing fragment, VIHN. Like in the case of albumin short models, peptides with an N-terminal amino group and a His in the third position often have a very strong affinity for metal ions. They form two five-membered and one six-membered chelate rings via coordination to the terminal NH_2_ group, two deprotonated amide nitrogens of residues in the second and third position, and N_δ_ from His in the third position to give the typical {N_Im_, 2N^−^, NH_2_} coordination mode [16]. In the case of Ni-VIHN complex, its stability constant was determined by CD measurements, since the rate of the reaction was very slow and the usual potentiometric method could not be applied. The low-spin, diamagnetic, square planar complex formed at pH above 8 appeared to be very stable, being its stability constant, measured as log K*, two orders of magnitude higher than those found for similar complexes bearing only glycine residues, as reported in Table 1 [17].

As a matter of fact, the stability constants are often expressed as their log values, and log K* represents a stability constant corrected for protonation of the ligand: log K* = log β(MH_j_L) − log β(H_n_L) for the equilibrium: M + H_n_L = MH_j_L + (n–j)H^+^. Values of equilibrium constants of actual complex formation as K* can be useful to compare the stability of similar species with analogue ligands when coordination comes from deprotonation. 

**Table 1:** log K* (4N) values of Ni(II) complexes of comparable XYH peptides.

Peptide	Protein	Ref.		logK *	Coordination sphere
Boc-AGGH		[18]	−30.02		{N_Im_, 3N^−^}
Ac-ELAKHA-Am	Histone H2B	[19]	−28.87		{N_Im_, 3N^−^}
Ac-IQTAVRLLLPGELAKHAVSEGTKAVTKYTSSK-Am	Histone H2B	[20]	−28.83		{N_Im_, 3N^−^}
Ac-AKRHRK-Am	Histone H4	[21]	−28.70		{N_Im_, 3N^−^}
Ac- SGRGKGGKGLGKGGAKRHRKVL -Am	Histone H4	[22]	−28.67		{N_Im_, 3N^−^}
Ac-TESHHK-Am	Histone H2A	[23]	−28.58		{N_Im_, 3N^−^}
Ac-TEAHHK-Am	Histone H2A *	[23]	−28.41		{N_Im_, 3N^−^}
Ac-TESHAK-Am	Histone H2A *	[23]	−28.23		{N_Im_, 3N^−^}
Ac-AK(Ac)RHRK(Ac)V-Am	Histone H4	[22]	−28.20		{N_Im_, 3N^−^}
Ac-TESAHK-Am	Histone H2A *	[23]	−28.18		{N_Im_, 3N^−^}
Ac-TRSRSHTSEGTRSR-Am	Cap43	[24]	−28.16		{N_Im_, 3N^−^}
Ac-TYTEHA-Am	Histone H4	[25]	−27.92		{N_Im_, 3N^−^}
Ac-TASHHK-Am	Histone H2A *	[23]	−27.26		{N_Im_, 3N^−^}
				
NH_2_-GGHistamine		[26]	−22.65		{N_im_, 2N^−^, NH_2_}
NH_2_-GGH		[15]	−21.81		{N_im_, 2N^−^, NH_2_}
NH_2_-SAHK-Am	Histone H2A *	[27]	−21.80		{N_im_, 2N^−^, NH_2_}
NH_2_-VIHN	Des-Angiotensinogen	[2]	−19.75		{N_im_, 2N^−^, NH_2_}
NH_2_-DAHK-Am	Albumin	[15]	−19.48		{N_im_, 2N^−^, NH_2_, O^-^}
NH_2_-RTHGQSHYRRRHCSR-Am	Protamine HP2	[28]	−19.29		{N_im_, 2N^−^, NH_2_}
NH_2_-RTHGQ-Am	Protamine HP2	[28]	−19.23		{N_im_, 2N^−^, NH_2_}
NH_2_-SHHK-Am	Histone H2A	[27]	−19.14		{N_im_, 2N^−^, NH_2_}

* modified sequence of Histone H2A.

A model structure of the Ni-VIHN species was obtained via a series of DQF-COSY, TOCSY and ROESY experiments, and it is shown in Figure 1b. From the calculated model it becomes evident that, while the Asn_4_ side chain does not seem to interact with the metal nor with the rest of the peptide, an ordering of the side chains of Val_1_ and Ile_2_ was present, with the formation of a hydrophobic barrier which was able to shield one side of the coordination plane from the bulk of the solution. The stabilities of Ni(II) peptide complexes depend on the rate of dissociation, which could require attack of water or H^+^ on one of the Ni(II)-amide nitrogen bonds [29,30]. In this case, the hydrophobic shielding from Val and Ile side chains can hinder the replacement of amide nitrogens with water molecules from above the plane of the complex, leading to a decrease in dissociation rate and increase in stability. It has been previously noted that hydrophobic shells around hydrophilic ligands appear to enhance metal binding to proteins [31,32].

### 2.3. Histones

#### 2.3.1. Histone H4

In a similar way we verified that nickel binding to the tail of histone H4 may organize the side chains around the metal centre. Furthermore, nickel coordination promoted a reorganization of the whole H4 tail so that an increase of the α-helical content was observed, leading to a shortening of the tail itself. It had been shown that histone H4 needs to be in its elongated form to accommodate into the specific recognition cleft on the HAT1-AcCoA complex and be normally acetylated for DNA transcription. Its nickel-promoted wrapping and shortening, therefore, is able to disrupt this mechanism, impairing the normal transcription route [33,34].

With a series of mono- and two-dimensional (NOESY and TOCSY) experiments, we had been able to assign all the resonances and to detect enough NOE cross-correlations to calculate a structural model for the Ni(II) complex with the 30-amino acid H4 tail, the ends blocked Ac-SGRGKGGKGLGKGGAK_16_R_17_H_18_RKVLRDNIQGIT-Am fragment [35].

The study was carried out at high pH values so as to allow the formation of the diamagnetic 4N {N_Im_, 3N^−^} complex. No signals were detected for the labile HN amidic protons under these conditions due to their fast exchange with water. In this way, the lack of HN basic meaningful NOE constraints made the structural determination of the whole complex not fully solved. However, significant NOEs were clearly detected for the residues directly involved in the complex formation and in its close proximity (A_15_K_16_R_17_H_18_R_19_), and this allowed determining a well-resolved restricted structure. The remaining part of the peptide was not completely characterized, showing nevertheless remarkable effects on the conformation caused by metal binding. NOESY cross-correlations were used as input data for structure calculations. The 20 best converged structures with lowest overall energy were chosen, and their average is shown in Figure 1c.

What emerged from this study was that as expected, Ni(II) at sufficiently high pH formed a square planar 4N complex with the involvement of His_18_ residue, together with three deprotonated amide nitrogens from the backbone, belonging to His_18_, Arg_17_ e Lys_16_, respectively. In addition, the involvement of Arg and Lys side chains was evidenced from the changes of their chemical shifts that clearly resembled those recorded for the albumin-Ni(II) complexes. In fact, Lys_16_ showed a strong up-field shift for its α proton after nickel binding, while all the remaining protons exhibited a neat down-field shift, according to the order ε < δ < γ < β. Furthermore, NOESY spectra showed a set of cross-peaks between the histidine H_ε1_ and all the aliphatic protons of Lys_16_ side chain. It thus seemed evident that Lys_16_ pointed directly towards the histidine residue passing over the coordination plane. Also in this case, the effect of both the metal and the ring current of the imidazole moiety played a role in determining the chemical shift changes observed for the lysyl protons.

From the analysis of the chemical shifts for the Ni(II)-peptide complex further remarkable information have been found. In fact, the neighbouring Arg_19_, which was not part of the coordination site, was anyway affected by nickel binding. The clear difference between the relative shifts of its two β protons may be due to a decrease in the conformational freedom of its side chain after coordination, a fact that was observed for all the different side chains in almost all the nickel complexes reported in this review. The structural model calculated for this H4-peptide Ni(II) complex showed the organization of the side chains around the metal ion where Lys_16_ and Arg_17_ protrude above the coordination plane while Arg_19_ extends below it, inducing an effective shield on both sides of the plane.

#### 2.3.2. Histone H2A

Amongst all histones, H2A is the only one to have two adjoining histidines within its sequence, an interesting feature that was examined in a number of papers. Some of these studies, carried out on small peptides containing the double histidine motif, showed that Ni(II) ions were not only able to bind to these fragments but also to cleave them, under right conditions, thanks to the assistance of a neighbouring serine [36,37]. The resulting cleaved peptide, blocked at its C-terminal end, SHHK-Am, together with its monohistidine analogue SAHK-Am, reported for comparison, were carefully studied for their interaction with Ni(II) and showed that both tetrapeptides can strongly bind Ni(II) ions, leading to square-planar complexes with 4N {N_Im_, 2N^−^, NH_2_} coordination above pH 10.0 [27].

The log K* value for Ni–SHHK complex is about two orders of magnitude higher than that found for Ni–SAHK complex (Table 1). This might be due to the effect of the positioning of the free imidazole ring, in the Ni–SHHK species, above the coordination plane, as suggested by spectroscopic evidence and theoretical predictions. 

Both 1D and 2D NMR experiments, flanked by potentiometric measurements and UV-Vis spectra, allowed for the full characterization of the complexes. The 4N square planar diamagnetic species appeared thus to be formed by the -NH_2_ group at the N-terminus, the imidazole nitrogen of His_3_, and the deprotonated amide nitrogens of the two residues in between (Ser_1_ and His_2_, or Ser_1_ and Ala_2_, respectively), as shown by the variations of their proton resonances. On the other hand, Lys_4_ did not participate in the coordination but the appearance of two new signals in the spectra of both complexes indicated the approaching of its side chain towards the bound imidazole ring, as was also reported in previous studies [15]. The most striking and uncommon aspect, instead, derived from the shifts of the imidazole proton signals in the uncoordinated His_2_ residue. The different interaction of the two imidazole rings with the metal ion is evidenced by the fact that His_2_ showed lower relaxation times for its imidazole protons [C_δ2_–H: 6 ms, C_ε1_–H: 4 ms] compared to those of His_3_ [C_δ2_–H: 93 ms, C_ε1_–H: 71 ms], thus explaining the found broad signals of His_2_ protons. Furthermore, while the ring protons of His_3_ underwent up-field shift upon nickel coordination, those of His_2_ shifted in the opposite direction. It is known from the literature that Ni(II) binding to an imidazole ring positioned on the equatorial plane, like in the case of His_3_, results in a shielding of the imidazole signals [21,23,24,38], so the down-field shift of His_2_ ring protons should mean a different interaction with the metal center. A possible explanation of this unusual behaviour may be due to the intramolecular interaction of the free imidazole electron ring cloud with the dz^2^ orbital of Ni(II) ions, leading to the deshielding of the imidazole protons. The positioning of this unbound ring parallel or closely parallel to the coordination plane in the Ni(II) complex with SHHK could protect one side of the coordination plane and seems to induce the extra stability as compared to the analogous SAHK complex.

The structural models for the two complexes were obtained through DFT theoretical calculations and showed a good agreement with the NMR-inferred structure, apart from the unbound His_2_ imidazole ring, which in this case is not parallel to the coordination plane but forms an angle of 65° with it (Figure 1d). This discrepancy must be due to the gas phase assumption for these calculations.

#### 2.3.3. Histone H2B

Histone H2B has in its sequence two possible sites for nickel binding, which were both extensively studied for their coordination behaviour. The first one is located in the histone-fold domain (63 to 93 residues), and its model peptide was chosen as the 31-amino acid Ac-NSFVNDIFERIAG_13_EASRL_18_A_19_H_20_YNKRS_25_TITSRE-Am fragment and studied towards Ni(II) complexation by multidimensional NMR spectroscopy (1D, 2D TOCSY, NOESY and ^13^C-HSQC) at pH 10.3, where maximum formation of the diamagnetic species was achieved [39].

At this pH, the labile amidic proton signals are lost and a well resolved solution structure of the Ni(II) complex was determined only for the 13 amino acid fragment, G_13_EASRL_18_A_19_H_20_YNKRS_25_, close to the metal center, that is the residue giving NOE cross-correlations in the NOESY spectra. NMR data showed that the coordination involved, as in histone H4, the imidazole nitrogen of His_20_ and the three adjacent amide nitrogens of His_20_, Ala_19_ and Leu_18_, respectively, in the well known 4N {N_Im_, 3N^−^} coordination mode. The side chains of other residues, anyway, showed the effects of the presence of Ni(II) ions: the aromatic ring of Tyr_21_ seems to place itself below the coordination plane, while the side chains of Arg_31_ and Glu_30_ seem to approach the metal too. The ordering of Leu_18_ and Ala_19_ side chains above the upper side of the coordination plane creates a hydrophobic fence that, together with the new position of Arg_17_, Tyr_21_ and Arg_24_ on the lower one, could be relevant to the complex stability, limiting the access of water molecules to the binding site. Furthermore, the location of the Tyr_21_ aromatic ring, close to the Ni(II) ion, can add extra stability to the complex via a possible interaction of the negative partial charge of the phenolic oxygen on the tyrosine with the positive part of the electrostatic potential generated by the nickel complex.

Another binding site in histone H2B may be found in its C-terminal tail, more specifically in the region around His_109_. For this reason, also the H2B_94-125_ fragment comprising peptide Ac-IQTAVRLLLPGE_12_LAKH_16_AVS_19_EGTKAVTK_27_YTSSK-Am has been examined towards Ni(II) binding [20]. This peptide incorporates a part of the α3 helix of the histone fold domain and the C-terminus [40], so that this portion of histone H2B should be easily accessible to nickel ions and is considered as one of the most likely binding sites within the whole protein. 

A well-resolved structure for the 4N {N_Im_, 3N^−^} square planar nickel complex including residues E_12_LAKH_16_AVS_19_ was obtained on the basis of the NOE connectivities recorded in two-dimensional NMR experiments. TOCSY, NOESY, ROESY and ^13^C-HSQC techniques have been employed to elucidate the details of nickel binding, while assignments on the free peptide have been facilitated by the use of ^1^H-^15^N-HSQC and 3D ^1^H-^1^H-^15^N-NHHa experiments.

Ni(II) coordination to the peptide caused mainly shifts of the resonances belonging to His_16_, Lys_15_, and Ala_14_, confirming that this is the portion of the peptide mostly involved in the coordination process through the imidazole nitrogen from His_16_ and three deprotonated amide nitrogens from the backbone, belonging to His_16_, Lys_15_ and Ala_14_, respectively.

As predictable, minor shifting of ^1^H- and ^13^C-NMR resonance signals was observed also for the neighbouring residues Glu_12_, Leu_13_, Ala_17_, Val_18_, Ser_19_, and Glu_20_, suggesting that also the magnetic environment of their nuclei is modified upon nickel binding. What was less expected was again the observation of severe changes in the resonances of very far away residues, those composing the C-terminal tail fragment Lys_27_-Tyr_28_-Thr_29_-Ser_30_-Ser_31_-Lys_32_, as confirmed by the cross-peaks evidenced in the ROESY and NOESY spectra. The involvement of remote residues in the coordination sphere contributes to a further stabilization of the complex itself, again through the phenolic oxygen of Tyr_28_ and the approach of Lys_27_ side chain to the binding site, as suggested by its α-proton up-field shift together with a down-field shift for all the remaining aliphatic protons (β > γ > δ > ε). It is thus evident the participation of different side chains to the stabilization of the complex, starting from the residues close to the central ion, Ala_17_ and Val_18_, which build up a hydrophobic fence above the plane, and the bulky aliphatic group of Leu_13_ below it; the approach of the distant Lys_27_-Tyr_28_-Thr_29_-Ser_30_-Ser_31_-Lys_32_ segment, covering up the whole coordination centre, contributes to form an effective shield against the bulk of the solution.

It is possible to conclude that, if such dramatic structural alterations are able to occur also under physiological conditions, it is highly probable they may interfere with the histone’s physiological role and particularly with the ubiquitination process, taking place at Lys120, one of the possible mechanism with which nickel is able to disrupt the normal transcription functions and result in carcinogenesis.

### 2.4. Prion Proteins

Prions have been extensively studied for metal binding with different cations, and they seem to have a particular affinity and be mainly involved in copper interaction. Nevertheless, Ni(II) was used as a diamagnetic probe to unveil the details of metal coordination to this protein outside the so called “tandem repeat”, which is known for being the effective copper binding site. Instead, a relatively conserved region in chickens and humans called the “amylodogenic region” was chosen, where a promising histidine residue was present. NMR experiments were then carried out on Ac-GGS_4_Y_5_H_6_N_7_QKP-Am, and especially NOESY experiments allowed for the collection of information on the metal complex structure in solution, since the detection of inter-residues NOE provided the proton-proton distances used in molecular dynamics programs (DYANA) to calculate the complex structural model [41]. This has been restricted only to the residues directly involved in metal coordination, due to the lack of any particular effects (chemical shift differences and NOE contacts) on the first two glycines and on the last three residues (Gln_8_, Lys_9_ and Pro_10_) which showed to be completely unperturbed by the metal ion. 

Also in this case, the complex is of 4N {N_Im_, 3N^−^} type, where imidazole nitrogen of His_6_ acts as the nickel anchoring site and the three remaining donors are the deprotonated amidic nitrogens from the backbone (His_6_, Tyr_5_ and Ser_4_, respectively).

The structure reported in Figure 1e showed a well defined Ni(II) binding site and revealed the proximity of Tyr_5_ ring to the metal ion. Such proximity, consistent with the large down-field shifts of Tyr aromatic protons, might play a key role in stabilizing the metal binding site.

### 2.5. Protamine

Protamines are small, arginine-rich, nuclear proteins that replace histones late in the haploid phase of spermatogenesis and are believed essential for sperm head condensation and DNA stabilization. They may allow for denser packaging of DNA in spermatozoon than histones, but they must be decompressed before the genetic data can be used for protein synthesis.

Mammals possess two classes of protamines, P1 and P2. The former, rich in arginine and cysteine, but not histidine, is expressed in all mammals, while the latter, which also contains histidine, has been detected in just a few mammalian species, including mice and humans.

Human protamine contains a metal-binding domain, the N-terminal tripeptide Arg-Thr-His, whose generic sequence with a histidine at the third position is similar to those encountered in several human peptide hormones and proteins, like in serum albumin (HSA). This short sequence is known to be specific for Cu(II) and Ni(II) binding [42].

It has been shown [43,44] that Ni(II) underwent activation upon binding to HP2_1-15_ peptide and became a DNA cleavage agent, so that this reactivity could be put in close relation with paternally transmitted transgenerational carcinogenesis in children of welders and machinists, professionally exposed to nickel-containing dusts and fumes [45].

A solution structure of the Ni(II) complex with the N-terminal pentadecapeptide (HP2_1–15_: R_1_T_2_H_3_G_4_Q_5_S_6_H_7_Y_8_RRRHCS) of human protamine HP2 was elucidated through a set of 1D and 2D ^1^H-NMR techniques (*i.e*., TOCSY, NOESY e ROESY) and molecular modeling [43]. From two-dimensional cross correlations, 52 inter-hydrogen constraints were obtained, including 16 inter-residual ones, which were used in structure determination using X-PLOR [46].

NMR assignments allowed establishing that the peptide is effectively unordered in solution, but upon nickel addition dramatic changes are observed. Cross-peaks appeared in the NOESY spectra among the resonances of Arg_1_, Thr_2_, and His_3_ residues as a result of the formation of a square-planar complex with Ni(II) ion, in the usual 4N {N_Im_, 2N^−^, NH_2_} fashion. Three histidines are present in the peptide, but only one, His_3_, is involved in metal coordination, the other two being affected only by an alteration in their electrostatic environment due to conformational changes after nickel binding, as shown by the sign of their chemical shift difference in the bound state and the absence of significant NOEs. The inter-residual cross-peaks can be seen also, among others, for signals of Arg_1_, Gln_5_, Tyr_8_, and Arg_15_ side chains. It appeared clear from NMR data and confirmed by all calculated conformers that the Tyr_8_ ring is located close to the Ni(II) coordination site. 

The geometry of the N-terminal nickel complex at the Arg_1_-Thr_2_-His_3_ sequence in the calculated model was restrained to the geometry of [Ni(II)(glycyl-glycyl-α-hydroxy-d,l-histamine]^.^3H_2_O complex, known from crystallographic measurements [47]. The conformers obtained were subdivided into three families, based on the details of the position of the ring versus the Ni(II) coordination plane and they all shared a unifying feature: the Tyr_8_ ring oxygen, carrying a partial negative charge, interacts with the positive part of the electrostatic potential generated by the nickel complex. This interaction, which can be interpreted as a kind of a salt bridge, should not be expected to be a major stabilizing force of the overall peptide conformation, but some stabilizing contribution cannot be excluded.

Finally, a striking double-loop conformation was found as a consequence of the interaction of Tyr_8_ aromatic ring with the terminal Arg_15_, by an unexpected long-range conformational effect exerted by nickel on this model peptide. All five positively charged arginine side chains tend to locate on one side of the molecule, making it possible to efficiently contact with the DNA double helix. All these structural changes, induced indirectly by Ni(II) coordination, indicate a possible function of HP2 N-terminus as a metal-binding site.

### 2.6. Cap43 Protein

Nickel, amongst other 12 different metals, specifically induces a cytoplasmatic protein called Cap43 which is overexpressed in a number of cancer cells. Its role, though not completely clarified, has been often connected to hypoxia [48,49,50,51,52].

Its most interesting feature is a monohistidinic decapeptide, TRSRSHTSEG, which is repeated consecutively three times at the C-terminus. We have already shown that Ni(II) is able to efficaciously and multiply bind to this sequences, since every single decapeptide fragment can anchor one metal ion. So, a detoxification role for Cap43 cannot be excluded. 

We have studied the details of nickel coordination to the three-repeat fragment by a range of potentiometric and spectroscopic experiments, but it was NMR that allowed us to build a structural model for the peptide-Ni(II) complex [52].

This study revealed that each decapeptide segment coordinates nickel in an independent way, so that the minimal 10-amino acid model, T_1_R_2_S_3_R_4_S_5_H_6_T_7_S_8_E_9_G_10_, may be used to describe the behaviour of the whole 30-amino acid fragment. At relatively high pH values (above 9) Ni(II) forms its square planar diamagnetic complex whose features have been unveiled by both mono- and two-dimensional NMR techniques. Again, the complex is of 4N {N_δIm_, 3N^−^} type, with His_6_, Ser_5_ and Arg_4_ as the involved residues. TOCSY, NOESY, ROESY and ^1^H-^13^C HSQC experiments were used to collect data for the assignment of all the resonances and calculation of the structural model (Figure 1f) from the recorded two-dimensional cross-correlations. 

Structural changes in the conformation of the peptide with organized Arg_4_ and Thr_7_ side chain orientation promoted by nickel coordination were detected. It is interesting to note that Thr_7_ side chain, although not involved in the metal complexation site, is located very close to the coordination centre, as confirmed by the size of its γ-protons shifts, while perturbations of its proton and carbon resonances are clearly visible in both 1D and 2D COSY and TOCSY spectra. Thr_7_ side chain can approach one of the axial positions in the coordination plane, while Arg_4_ side chain approaches the other. This is also shown by the down-field chemical shift effect on its side chain protons, which are affected according to the β *>* γ *>* δ order, while the H_α_ moves in the opposite direction since shielded by the electron density deriving from the amide deprotonation. This deshielding effect along the Arg_4_ side chain might be due to the influence of both the metal and the histidine ring current while it extends below the coordination plane.

Thus, it appears evident that two side chains, one from Thr_7_ and one from Arg_4_, with their location above the two sides of the coordination plane also in this case may organize themselves as to protect the complex from the action of water molecules. 

## 3. Discussion

It is well known that NMR is one of the election techniques for studying metal complexes in solution, allowing the determination of reliable complex structures. It is a versatile and fast method which is able to supply information on different aspects of metal complexes, in an environment which can be tuned to mimic the biological one in order to give details that for example crystallography cannot always afford.

Nickel is an essential trace element involved in the metabolism of several species of archea, bacteria and plants [53]: the urease enzyme, for instance, contains up to 12 nickel atoms [54]. Although a physiological role has been suggested for higher organisms and animals, it is still debated whether nickel could be essentials also for humans [53]. Nevertheless, human cells possess Ni-specific receptors [55,56], so a biological function for this metal could not be fully denied. Even so, its noxious effects are well documented and studied [53] and the mechanisms through which they are exerted are slowly being unveiled, an important contribution given by the papers here examined. 

It appears evident that these mechanisms are based on nickel interactions with its cellular targets, such as the proteins mentioned throughout this review. The stronger the interactions, the most pronounced the effects, since a labile complex is easily dismantled by a series of equilibria as those reported in Scheme 1. So, any extra stabilization of the nickel complexes deriving from the side chains shielding around the metal site can be crucial for the fate of the complex itself, granting it a long lifetime and then the chance to exert its action inside the organism.

As reminded in the introduction, the complex stability is reflected by its formation constant, K, whose expression may be corrected to K* when deprotonation is involved. Ks may be calculated both through potentiometric and spectroscopic methods, the former being preferred when complexes are soluble and kinetics fast enough. The evidence of an extra stabilization, due to the shielding supplied by the side chains organization, can be seen from the Ks* values. They have been collected in Table 1 for all the examined complexes in the same coordination mode, both {N_Im_, 2N^−^, NH_2_}, {N_Im_, 2N^−^, NH_2_,O^−^} or {N_Im_, 3N^−^}. It has been found out that the species showed a higher stability (at least one or two orders of magnitude depending on the complex) compared to analogous species where no bulky side chains were present, bearing, for example, glycines instead of lysines, arginines, tyrosines or threonines. 

**Figure 2:**
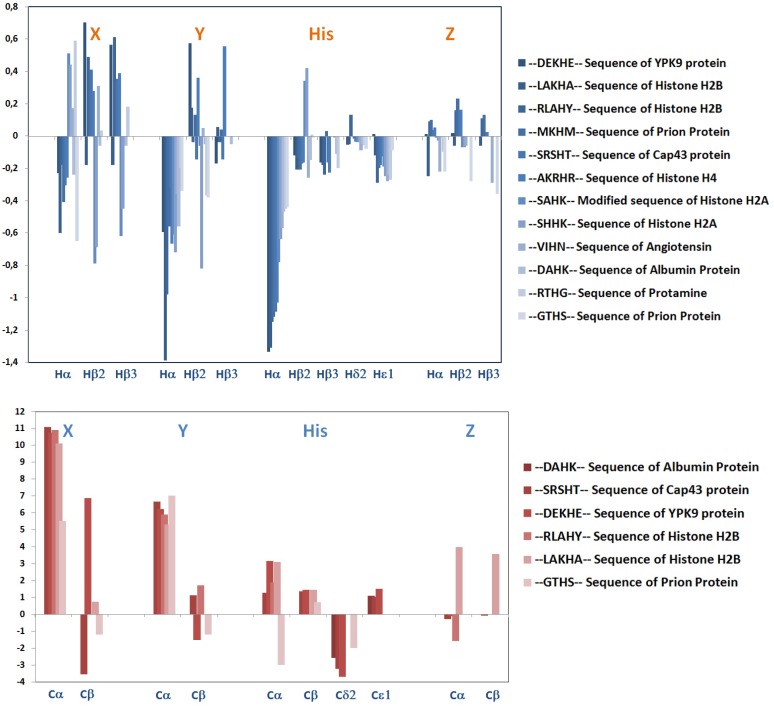
Plot of ^1^H- and ^13^C-NMR chemical shift variations (Δδ = δ_holo_ − δ_apo_, ppm) of selected analogous sequence with Ni(II) bound in a 4N based coordination mode.

Although the different “environment” around the nickel anchoring site and the different coordination fashion, due to the variety of peptides, make this series of nickel complexes not perfectly homogeneous, nevertheless a general trend can be found in the chemical shifts of protons and carbons close to the coordination site, (H_α_ and H_β_) and (C_α_ and C_β_), as shown in Table 2 and in the plot reported in Figure 2. 

It seems clear that, in the generic sequence X-Y-H-Z, the histidine residue is, as expected, the most affected one, with a general shielding of all its protons in almost all cases studied, due to the effect of amide deprotonation and metal coordination. This fate is shared by the H_α_ proton of the preceding residue Y and even in a lesser extent also for X, whose amide NHs have been deprotonated to allow the formation of the square planar complex. As for the H_β_ protons, their up-field or down-field shifts depend on the relative positioning of the side chains within the metal complex, so the trend is not regular and any prediction would not be feasible. Still, the trend in the residue closer to the histidine, Y, is more regular in comparison to the further one, X. As for Z, on the other side of the sequence, the Δδ is even less marked, and again it corresponds to a more or less involved participation to the complex formation and to its side chain relative location around the metal centre.

Although data for the carbon shifts are not available or incomplete for all the sequences here analyzed, the recognition of comparable Δδ values appears to be more relevant, in particular for all the carbon nuclei of the histidine residues. Almost in all cases, they share the same trend in the Δδ difference.

In a similar way as for the side chain protons, also for their carbon resonances the Δδ values are more variable, depending on the space localization and relative electronic environment, which forbids any generalization.

Table 2^1^H- and ^13^C-NMR chemical shift variations (Δδ = δ_holo_ − δ_apo_, ppm) of selected analogous sequence with Ni(II) bound in a 4N based coordination mode.

**Table:** 

XY-His-Z sequence	Protein	Ref.		X			Y				His				Z	
			Hα	Hβ2	Hβ3	Hα	Hβ2	Hβ3	Hα	Hβ2	Hβ3	Hδ2	Hε1	Hα	Hβ2	Hβ3
DEKHE	YPK9	*	−0.23	0.70	0.56	−0.59	0.57	−0.17	−1.33	−0.12	−0.16	−0.06	0.01	0.01	0.02	0.00
LAKHA	Histone H2B	[20]	−0.60	−0.18	−0.18	−1.39	0.18	0.06	−1.31	−0.21	−0.18	−0.05	−0.12	−0.25	−0.06	−0.06
RLAHY	Histone H2B	[39]	−0.18	0.49	0.61	−0.98	−0.04	−0.04	−1.15	−0.14	−0.24	0.13	−0.29	0.09	0.16	0.11
MKHM	Prion Protein	[57]	−0.41	0.41	0.34	−0.56	0.13	0.04	−1.12	−0.21	0.03	0.00	−0.20	0.10	0.23	0.13
SRSHT	Cap43	[52]	−0.31	0.41	0.35	−0.32	−0.15	−0.15	−1.09	−0.17	−0.17	−0.02	−0.18	0.04	0.16	
AKRHR	Histone H4	[35]	−0.26	0.28	0.39	−0.67	0.36	0.55	−1.03	−0.17	−0.23	−0.04	−0.13	0.05	0.16	0.03
SAHK	Histone H2A	[27]	0.51	−0.79	−0.62	−0.61	-0.06		−0.78	0.34		−0.04	−0.19	−0.01	−0.07	
SHHK	Histone H2A	[27]	0.44	−0.69	−0.45	−0.72	-0.82		−0.64	0.42		−0.04	−0.25	−0.03	−0.07	
VIHN	Angiotensin	[2]	0.17	0.31		−0.36	0.05		−0.57	−0.26	−0.01	−0.09	−0.28	−0.22	−0.06	−0.29
DAHK	Albumin	[6]	−0.24	−0.06	−0.06	−0.56	−0.05	−0.05	−0.47	−0.15	−0.11	−0.05	−0.24			
RTHG	Protamine	[43]	0.59	0.03	0.18	−0.20	−0.37		−0.45	0.01	−0.20	−0.06	−0.27	−0.10		
GTHS	Prion	[41]	−0.65			−0.34	−0.38		−0.44	−0.01		−0.08	−0.09	−0.22	−0.28	−0.36

**Table:** 

XY-His-Z sequence	Protein	Ref.		X			Y				His				Z	
			Cα		Cβ	Cα		Cβ	Cα	Cβ		Cδ2	Cε1	Cα		Cβ
DEKHE	YPK9	*	10.77		6.87	6.21		−1.54	3.17	1.45		−3.70	1.51	−0.11		−0.03
SRSHT	Cap43	[52]	11.07		−3.55	6.67		1.11	1.26	1.36		−3.23	1.02	−0.28		−0.09
RLAHY	Histone H2B	[39]	10.9		NA	5.9		1.7	1.9	NA		NA	NA	−1.6		NA
LAKHA	Histone H2B	[20]	10.1		0.73	5.31		NA	3.09	1.45		NA	NA	3.98		3.58
DAHK	Albumin	[6]	NA		NA	NA		NA	NA	NA		−2.59	1.1	NA		NA
GTHS	Prion	[41]	5.5		−1.2	7		−1.2	−3	0.7		−2	0	0		0

* Unpublished data. For information about –DEKHE- sequence of YPK9 protein see Refs. [58,59].

## 4. Conclusions

Although the stability of a metal complex derives from many factors, among the others the nature of the donor atoms present on the coordinating peptide, the orientation and organization of the side chains around a metal centre showed to be of remarkable importance in the study of such complexes with peptides and proteins, as evidenced by the number of papers here examined. We have learned that the side chain ordering around the coordination plane can shield it from the attack of water molecules, which represent the most abundant active species in solution, as well as from other small coordinating compounds present in the cellular environment, often by building up a hydrophobic fence. We have also learned that the interactions of the metal centre with the peptide side chains can hinder their conformational freedom by blocking them in a more confined disposition compared to the unbound peptide. If such structural alterations may occur under physiological conditions on the “parent” proteins, it is highly possible they may interfere with several biological processes, for example in the correct folding processes, in the assembly of specific receptor binding sites as well as in the catalysis at the metal site; for all these reasons, they should always be taken into account when dealing with functioning and stability of such metal species.
